# Nonclassic Congenital Adrenal Hyperplasia Metabolic Resolution Post Roux-en-Y Gastric Bypass and Associated Weight Loss

**DOI:** 10.1210/jcemcr/luae018

**Published:** 2024-02-23

**Authors:** Karina G Romo, Sharon W Shu, Qasim Z Iqbal, Gabriel I Uwaifo

**Affiliations:** Division of Endocrinology, Cedars-Sinai, Los Angeles, CA 90048, USA; University of Queensland-Ochsner Clinical School, Brisbane, QLD 4072, Australia; Endocrinology Division, Indiana University School of Medicine, Indianapolis, IN 46202, USA; Endocrinology Division, Department of Medicine, Southern Illinois University School of Medicine, Springfield, IL 62702, USA

**Keywords:** nonclassic congenital adrenal hyperplasia, Roux-en-Y gastric bypass, morbid obesity, class III obesity, bariatric surgery, massive weight loss

## Abstract

Nonclassic congenital adrenal hyperplasia (NCCAH) is characterized by mild cortisol deficiency, excess androgens and adrenocorticotropin (ACTH) production, and often with various features of dysmetabolic syndrome. Elective bariatric surgery is one of the most effective long-term management strategies for severe obesity. Our case presents a 34-year-old woman with symptomatic NCCAH and class III obesity who status post Roux-en-Y gastric bypass (RYGB) had significant weight loss with metabolic resolution of NCCAH, and no longer required glucocorticoid (GC) therapy. At 11 months post operation and off GC therapy, she had a weight deficit of approximately 160 pounds (72.57 kg) with continued metabolic resolution of NCCAH markers including ACTH, 17-hydroxyprogesterone, and androstenedione. Presently, GC therapy remains one of the few available treatments for symptomatic NCCAH; however, long-term GC therapy has the potential for various complications and side effects. Our case presents elective bariatric surgery as a potential and unique treatment option for patients with NCCAH with associated class III obesity. The exact pathophysiologic basis for this effect and its potential role in long-term management of appropriate NCCAH patients requires further study.

## Introduction

Congenital adrenal hyperplasia (CAH) encompasses a group of rare autosomal recessive disorders, most of which result from loss-of-function mutations of the cytochrome P450 (CYP) *21A2 (CYP21A2)* gene (1). *CYP21A2* codes for 21-hydroxylase, an important adrenal enzyme in the cortisol biosynthesis pathway. The classic type of CAH results from a near absence of 21-hydroxylase enzyme activity and typically presents in infancy or early childhood with ambiguous genitalia and/or salt-wasting. Meanwhile, patients with nonclassic congenital adrenal hyperplasia (NCCAH) retain 20% to 50% of 21-hydroxylase enzyme activity (2). Consequently, NCCAH patients are commonly asymptomatic; when symptomatic, NCCAH patients present later in life.

NCCAH is characterized by mild cortisol deficiency, disinhibited adrenocorticotropin (ACTH) production, androgen excess with elevated 17-hydroxyprogesterone (17-OHP), and often various features of dysmetabolic syndrome (3). Because cortisol deficiency is mild, symptomatic patients with NCCAH may present with signs of androgen access rather than glucocorticoid (GC) insufficiency (2). Symptoms of hyperandrogenism include acne, hirsutism, and menstrual dysfunction, including anovulation and subfertility. Although there is no cure for NCCAH, GCs are the mainstay of treatment for symptomatic patients; thus, guidelines for NCCAH treatment recommend GC for women with patient-significant symptoms of hyperandrogenism or infertility (4).

The effect of weight loss on symptoms and metabolic parameters for patients with NCCAH is unknown. There are currently no identified published case reports of patients with NCCAH who have had significant and sustained weight loss from lifestyle change alone. Regardless of CAH, elective bariatric surgery is one of the most effective long-term management strategies for class III (formerly morbid) obesity. Common types of bariatric surgery are the gastric band, gastric sleeve, and gastric bypass. The Roux-en-Y gastric bypass (RYGB) procedure consists of stapling a section of the upper stomach to create a small stomach pouch. The small intestine is then divided into 2 parts, with the upper Roux limb attached to the small stomach pouch and the lower biliopancreatic limb connected to the lower section of the small intestine, creating a Y-shaped configuration (5). By bypassing the majority of the stomach and upper small intestine, the RYGB reduces the amount of food that can be consumed and limits the absorption of nutrients, while also significantly altering the gut-derived hormonal milieu, ultimately resulting in profound weight loss. This case presents a woman with symptomatic NCCAH and complicated class III obesity who status post RYGB had significant weight loss associated with metabolic resolution of NCCAH and no longer required GC therapy.

## Case Presentation

A then 28-year-old nulliparous woman with well-managed hypothyroidism, thyroid nodular disease, dyslipidemia, and class III obesity initially presented to endocrinology on account of excessive coarse hair growth on her face, chest, lower abdomen and back, as well as secondary amenorrhea initially attributed to polycystic ovary syndrome (PCOS). The presumed PCOS was managed with oral contraceptive pill (OCP) therapy, spironolactone, and metformin. However, the patient had persistent menstrual irregularities with hopes of fertility and difficult to manage hirsutism requiring frequent cosmetic interventions including depilator creams and shaving.

## Diagnostic Assessment

Initial visit vital signs were notable for blood pressure of 128/86 mm Hg, pulse 91/min, height 5 feet 6 inches (167.64 cm), weight 324 pounds (146.964 kg), and body mass index (BMI) of 52.3. Physical examination showed central adiposity without active hirsutism (managed with cosmetic interventions). Pelvic ultrasound was unremarkable without typical PCOS appearance.

She was diagnosed with NCCAH after initial screening revealed elevated androstenedione 364 ng/dL (12.70 nmol/L) (reference range, 51-230 ng/dL; 1.78-8.03 nmol/L), testosterone 68 ng/dL (2.36 nmol/L) (2-45 ng/dL; 0.07-1.56 nmol/L), ACTH 50 pg/mL (11 pmol/L) (0-46 pg/mL; 0-10.12 pmol/L), and random 17-OHP level, 1275 ng/dL (38.63 nmol/L) (35-413 ng/dL; 1.06-12.5 nmol/L). A subsequent ACTH stimulation test revealed 1-hour values of 17-OHP 3490 ng/dL (105.7 nmol/L) (35-413 ng/dL; 1.06-12.5 nmol/L) and cortisol 19.1 µg/dL (526.9 nmol/L) (4.30-22.4 µg/dL; 118.6-617.97 nmol/L). Baseline laboratory values and trends are given in Table 1. There was no evidence of salt-wasting. Follow-up 21-hydroxylase genetic testing showed single-copy deletion *CYP21A2* and the pathogenic variant *p.V282L* in the remaining copy. Based on her elevated 17-OHP and androstenedione levels, her CAH was considered metabolically active.

**Table 1.** Summary of nonclassic congenital adrenal hyperplasia serum markers at selected time points

Serum labs (reference range)	Initial presentation (2017)	Prior to initiation of GC (2017)	2 y post-GC therapy (2019)	Interim pregnancy (2020)	6 mo post RYGB and off GC (2022)	11 mo post RYGB and off GC (2022)
17-hydroxyprogesterone (35-413 ng/dL35-413 ng/dL; 1.06-12.5 nmol/L)	1275 ng/dL*^b^* (38.63 nmol/L)	1756ng/dL*^b^* (53.20 nmol/L)	345 ng/dL (10.45 nmol/L)	1030 ng/dL*^b^* (31.21 nmol/L)	140 ng/dL (4.24 nmol/L)	189 ng/dL (5.73 nmol/L)
ACTH (0-46 pg/mL; 0-10.12 pmol/L)	50 pg/ml*^b^* (11 pmol/L)		33 pg/mL (7.26 pmol/L)	85 pg/ml*^b^* (18.7 pmol/L)	16 pg/mL (3.52 pmol/L)	10 pg/mL (2.2 pmol/L)
Aldosterone (0-39.2 ng/dL; 0-1087.41 pmol/L)	38.2 ng/dL (1059.67 pmol/L)		41.1 ng/dL*^b^* (1140.11 pmol/L)	30.2 ng/dL (837.75 pmol/L)	17.9 ng/dL (496.55 pmol/L)	16.1 ng/dL (446.61 pmol/L)
Renin (Na-depleted, upright: 2.9-24 ng/mL/h; 2.9-24 µg/L/h)			7.7 ng/mL/h (7.7 µg/L/h)	5.0 ng/mL/h (5.0 µg/L/h)	8.9 ng/mL/h (8.9 µg/L/h)	6.6 ng/mL/h (6.6 µg/L/h)
Cortisol 8 Am (4.30-22.40 μg/dL; 118.63-617.97 nmol/L)	17.9 µg/dL (493.83 nmol/L)					
Random cortisol (1.7-14.1 μg/dL: 46.9-388.99 nmol/L)			6.50 µg/dL (179.32 nmol/L)	11.90 µg/dL (328.30 nmol/L)	6.60 µg/dL (182.08 nmol/L)	11.20 µg/dL (308.99 nmol/L)
DHEA-sulfate (95.8-511.7 μg/dL; 2.59-13.82 µmol/L)	356.8 µg/dL (9.63 µmol/L)	254 µg/dL (6.86 µmol/L)	69.0 µg/dL*^a^* (1.86 µmol/L)	63.6 µg/dL*^a^* (1.72 µmol/L)	145.2 µg/dL (3.92 µmol/L)	114.3 µg/dL (3.09 µmol/L)
DHEA (1.330-7.78 ng/mL; 4.62-27.0 nmol/L)	2.72 ng/mL (9.44 nmol/L)	1.78 ng/mL (6.18 nmol/L)	2.90 ng/mL (10.06 nmol/L)	0.775 ng/ml*^a^* (2.69 nmol/L)	1.51 ng/mL (5.24 nmol/L)	1.149 ng/mL (3.99 nmol/L)
Testosterone (2-45 ng/dL; 0.07-1.56 nmol/L)	35 ng/dL (1.21 nmol/L)	68 ng/dL*^b^* (2.36 nmol/L)	56 ng/dL*^b^* (1.94 nmol/L)			13 ng/dL (0.45 nmol/L)
Androstenedione (51-230 ng/dL; 1.78-8.03 nmol/L)	143 ng/dL (4.99 nmol/L)	364 ng/dL*^b^* (12.7 nmol/L)			75 ng/dL (2.62 nmol/L)	33 ng/dL (1.15 nmol/L)
Estradiol (30-400 pg/mL; 110.13-1468.40 pmol/L)		68 pg/mL (249.63 pmol/L)				
Progesterone (5-20 ng/mL; 15.90-63.60 nmol/L)		4 ng/mL (12.72 nmol/L)				

Abbreviations: ACTH, adrenocorticotropin; DHEA, dehydroepiandrosterone; GC, glucocorticoid; RYGB, Roux-en-Y gastric bypass.

^*a*^Values less than the reference range.

^*b*^Values greater than the reference range.

## Treatment

The patient was prescribed GC therapy for symptomatic amelioration of the NCCAH features using hydrocortisone 10 mg 3 times a day. This treatment improved her hirsutism and decreased the time needed between cosmetic interventions for excessive hair growth. Additionally, regular menstruation resumed. While on GC, her BMI did increase to 57.08 over the next 15 months; however, the weight gain was most likely not related to GC since she was taking a physiologic replacement dose. Phentermine and topiramate were started. After a further 2 years of stable management on GC, the patient became pregnant unexpectedly. Phentermine and topiramate were discontinued. Metformin was also discontinued following normal gestational oral glucose tolerance tests. She remained on gestational GC and was lost to endocrine follow-up at this time.

Then, at age 33 years, 15 months postpartum, and 6 months’ status post RYGB, the patient returned to endocrinology care. The postpartum course had been complicated by substantial weight gain, with a maximum weight post delivery of 423 pounds (191.87 kg), which led her to seek elective bariatric surgery for long-term weight management. Post RYGB, physical examination was significant for a weight of 265.7 pounds (120.5 kg), representing a weight deficit of approximately 100 pounds (45.36 kg). Although she self-discontinued GC therapy for fear of postoperative weight gain, her amenorrhea resolved, and her hirsutism significantly improved with decreased appearance of coarse hair. Laboratory testing revealed metabolic resolution of NCCAH noted by normalization of 17-OHP 140 ng/dL (4.24 mmol/L) (35-413 ng/dL; 1.06-12.5 nmol/L) and androstenedione 75 ng/dL (2.62 nmol/L) (51-230 ng/dL; 1.78-8.03 nmol/L). Thus, hydrocortisone was held for another 6 months. Spironolactone and OCP therapy were resumed postpartum.

## Outcome and Follow-up

**Figure 1.**
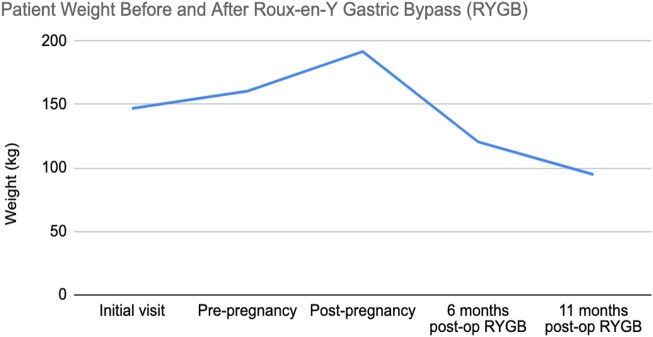
Patient weight trend in kilograms before and after Roux-en-Y gastric bypass (RYGB).

At 11 months post operation and off GC treatment, the patient weighed 209 pounds (94.8 kg), had a total weight deficit of approximately 160 pounds (72.57 kg), and a BMI of 34.39. Her weight trend before and after RYGB are shown in Fig. 1. She had continued control of hyperandrogenic symptoms with normal regular menstrual cycles. Laboratory values noted continued metabolic resolution of NCCAH with normal 17-OHP 189 ng/dL (5.73 nmol/L) (35-413 ng/dL; 1.06-12.5 nmol/L), androstenedione 33 ng/dL (1.15 nmol/L) (51-230 ng/dL; 1.78-8.03 nmol/L), testosterone 13 ng/dL (0.45 nmol/L) (2-45 ng/dL; 0.07-1.56 nmol/L), and ACTH 10 pg/mL (2.2 pmol/L) (0-46 pg/mL; 0-10.12 pmol/L). Thyroid function tests were euthyroid. Hydrocortisone was not restarted.

## Discussion

This case presents a patient with NCCAH and class III obesity who underwent RYGB that resulted not only in substantial weight loss, but also resolution of amenorrhea, improvement in hirsutism, and normalization of NCCAH laboratory markers; thus, GC treatment was not needed postoperatively. GC therapy is the standard for symptomatic management of menstrual irregularities, hirsutism, and infertility in NCCAH patients. However, long-term GC therapy has the potential for various complications and side effects, including cardiovascular consequences (such as blood pressure elevation and dyslipidemia), weight gain, and poor bone health (including osteopenia or frank osteoporosis induction). Of these, secondary cortisol insufficiency is a particular concern for NCCAH patients with unreliable baseline adrenal function (6). Therefore, it is preferable to keep GC doses low. Current strategies for minimizing GC include prescribing supplemental OCPs and spironolactone to address irregular menstruation and hirsutism, respectively; however, there are NCCAH patients whose symptoms are not controlled despite current GC minimizing strategies.

Expanding current strategies for minimizing GC use is especially relevant given the side effects discussed. Since weight loss and bariatric surgery are reported to be beneficial for patients with certain metabolic diseases such as diabetes and PCOS, it is worth exploring as a possible treatment for patients with CAH and obesity (7). It is known that following bariatric surgery, there are considerable physiologic responses to postsurgical change in gastrointestinal (GI) tract morphology, including reduced serum triglycerides and glucose, as well as increased postprandial adiponectin, glucagon-like peptide 1 (GLP-1), insulin, and insulin-like growth factor 1 (8, 9). Similar postsurgical kinetics and changes in metabolism may explain our patient's NCCAH remission.

This case report does have limitations. We do not have endocrinological data just before this patient underwent RYGB because she was lost to follow-up after pregnancy and before RYGB; consequently, the interim pregnancy is confounding. She was also restarted on spironolactone and OCP after pregnancy and continued post RYGB. These confounding medications may have contributed to improvement in her hyperandrogenic symptoms and menstrual regulation; however, these treatments had not improved her symptoms nor metabolic markers when she had taken them before pregnancy and bariatric surgery. Additionally, there are no data in this report or on literature review to say RYGB itself was the reason for metabolic resolution of NCCAH rather than associated weight reduction. We did not identify any published case reports of individuals with CAH who have had this degree of weight loss from lifestyle change alone or lifestyle with pharmacotherapy alone. Not only is CAH rare, but it is also uncommon to have patients lose and maintain weight loss to the degree of this patient (>100 pounds; 45.4 kg) by nonsurgical means, thus, to have the confluence of both CAH and lifestyle-induced massive weight loss is even more unlikely. However, we did identify 3 published case reports noting improvement in CAH post bariatric surgery, though no case series were found on this subject.

In the first case by Mallappa et al (10), the authors consider alterations in hydrocortisone pharmacokinetics as an influence on dramatic weight loss, improved insulin sensitivity, and improved fatty liver disease seen after sleeve gastrectomy in a young patient with CAH. To investigate this, they performed cortisol clearance studies on the patient that noted decreased volume of distribution and cortisol clearance post bariatric surgery. Thus, the GC dose was decreased by 34% and the patient had continued control of her hyperandrogenism 27 months post bariatric surgery (10). In a separate case report by Kalani et al (11), the authors note that bariatric surgery has recently been recognized to improve insulin resistance and ameliorate PCOS, and therefore may be beneficial in NCCAH as well. They report a case, similar to ours, of a young woman with NCCAH whose clinical and biochemical manifestations of hyperandrogenism and obesity significantly improved after RYGB while off her presurgery medications of metformin and pioglitazone. The third case, by Zatsepina et al (12), describes a patient with CAH complicated by insulin resistance and obesity on GC therapy who 7 months post laparoscopic sleeve resection achieved a “reduction in prednisolone dose by 25% and a decrease in body weight by 72.1% of overweight,” which serves as an example of treating severe obesity in CAH with bariatric surgery as a strategy to minimize GC use.

At this point, it is unclear if observed metabolic improvements following bariatric surgery in this case report are entirely weight loss driven or also the result of unique hormone changes of the GI-endocrine axis that are known post RYGB (9). Based on the paper by Mallappa et al (10), it may be the joint result of substantial weight loss, and hormonal and GI humoral changes that RYGB induces.

In combination with the 3 discussed cases, our case raises the possibility of employing elective bariatric surgery as a potential and unique treatment option for patients with NCCAH who have associated severe obesity. Additionally, the potential mechanism of altered GC pharmacokinetics post bariatric surgery raises the question of whether there may be a role for drugs that alter gut hormonal milieu, affect metabolism, and induce weight loss, such as GLP-1 receptor agonists, in treating NCCAH. The exact pathophysiologic basis for our observed effect and its potential role in the long-term management of appropriate NCCAH patients require further in-depth study.

## Learning Points

- Elective bariatric surgery is one of the most effective long-term management strategies for class III and complicated obesity including obesity in patients with NCCAH.
- Symptomatic and metabolic resolution of NCCAH may be achievable by weight loss post RYGB without the need for ongoing GC therapy.
- The potential mechanism of altered GC pharmacokinetics post bariatric surgery raises the question of whether there may be a role for drugs that alter the gut hormonal milieu, affect metabolism, and induce weight loss, such as GLP-1 receptor agonists, in treating NCCAH.

## Data Availability

Data sharing is not applicable to this article as no data sets were generated or analyzed during the current study.

## Abbreviations

17-OHP: 17-hydroxyprogesterone
ACTH: adrenocorticotropin
BMI: body mass index
CAH: congenital adrenal hyperplasia
*CYP21A2*: cytochrome P450 *21A2*
DHEA: dehydroepiandrosterone
GC: glucocorticoid
GI: gastrointestinal
GLP-1: glucagon-like peptide 1
NCCAH: nonclassic congenital adrenal hyperplasia
OCP: oral contraceptive pill
PCOS: polycystic ovary syndrome
RYGB: Roux-en-Y gastric bypass
